# Learning increases growth and reduces inequality in shared noisy environments

**DOI:** 10.1093/pnasnexus/pgad093

**Published:** 2023-03-22

**Authors:** Jordan T Kemp, Luís M A Bettencourt

**Affiliations:** Department of Physics, University of Chicago, 5720 S Ellis Ave #201, Chicago, 60637 IL, USA; Department of Ecology & Evolution, University of Chicago, 1101 E 57th St, Chicago, 60637 IL, USA; Mansueto Institute for Urban Innovation, University of Chicago, 1155 E 60th Street, Chicago, 60637 IL, USA

**Keywords:** growth, information, inequality, learning

## Abstract

Stochastic multiplicative dynamics characterize many complex natural phenomena such as selection and mutation in evolving populations, and the generation and distribution of wealth within social systems. Population heterogeneity in stochastic growth rates has been shown to be the critical driver of wealth inequality over long time scales. However, we still lack a general statistical theory that systematically explains the origins of these heterogeneities resulting from the dynamical adaptation of agents to their environment. In this paper, we derive population growth parameters resulting from the general interaction between agents and their environment, conditional on subjective signals each agent perceives. We show that average wealth-growth rates converge, under specific conditions, to their maximal value as the mutual information between the agent’s signal and the environment, and that sequential Bayesian inference is the optimal strategy for reaching this maximum. It follows that when all agents access the same statistical environment, the learning process attenuates growth rate disparities, reducing the long-term effects of heterogeneity on inequality. Our approach shows how the formal properties of information underlie general growth dynamics across social and biological phenomena, including cooperation and the effects of education and learning on life history choices.

**Significance**

Current approaches for studying wealth dynamics and inequality lack a foundational theory to derive growth rates from social behavior in unknown environments. Devising effective interventions to manage economic growth rates, financial instability, and population inequality remains therefore difficult. Here, we propose a general approach to this problem based on agent decision-making in noisy environments, using concepts of information and learning. We show that expanding learning reduces resource inequality over time, as more agents are able to tap opportunities in their environment. This perspective connects wealth dynamics to important behavioral and social phenomena such as the environmental determinants of learning and development, the influence of socioeconomic stratification and segregation, and information sharing, cooperation and resilience in the face of uncertainty.

## Introduction

Growth and inequality are fundamental general properties of biological and social complex adaptive systems (1). They are essential in human societies, where growth determines aggregate prosperity, and heterogeneity of growth across individuals has implications for opportunity and equity (2). Recently, richer data have enabled new approaches toward studying growth and inequality through the statistical dynamics of populations based on the behavior of forward-thinking agents. For example, we now have general answers connecting growth and redistribution models to specific standing levels of inequality (3–5). However, there remain general questions about how societies can promote long-term growth while controlling or mitigating inequality, and more broadly how agent behavior influences aggregate growth.

To address these questions, researchers have sought to better understand the non-linear dynamics of wealth distributions through quantitative modeling. These approaches include the generation and redistribution of incomes and costs among agents within model societies (6–9), and the derivation of long-term steady-state wealth distributions (3–5, 10, 11). In much of this work, agents representing individuals or households (often with life cycles) grow or lose wealth through a multiplicative (geometric) stochastic process. This modeling choice is well supported empirically and introduces a number of key parameters as an agent’s resources (or wealth), *r*, evolve exponentially with mean growth rate (over time), *γ*, fluctuate with standard deviation (volatility), *σ* (3, 12, 13), and vary across individuals of a population with standard deviation $\sigma_{\gamma }$ (14, 15). These parameters describe the statistical dynamics of wealth in heterogeneous populations and the emergence of inequality across various timescales.

The statistics of heterogeneous growth rates are particularly important, as they are the predominant drivers of inequality over long times (14). In such contexts, agents with higher average growth rates amass more relative wealth, reducing social mobility across populations. This phenomenon has been well known to economists, who have studied its emergence in models of elastic agent decision-making for goods exchanges (16–18), and its aggregate impacts via heterogeneous growth resulting from firm innovation (19) and natural resource abundance (20). Despite the impact of heterogeneity in stochastic growth systems, as observed both in models and in empirical data, we still lack a theoretical explanation for the origins of growth rates and volatilities compatible with stochastic geometric growth models. Specifically, we need a set of general principles and resulting statistical mechanics of agent decisions in stochastic environments to explain how differences in agent behavior result in heterogeneous growth. Such a theory would enable further study into not just how optimal agent decisions contribute to inequality in time but also across levels of social organization (21).

Recent developments in cognitive and ecological sciences can provide some valuable insights into the stochastic dynamics of agent behavior (22). Researchers using noisy decision-making models to explore child and adolescent development have recently rethought the process of human learning in terms of acquiring information through (active and passive) interactions with a knowable, but stochastic external environment (23–25). Similarly, ecologists have formulated natural selection, the process through which a genotype optimally leverages its environment’s structure to maximize population growth (fitness), as a (Bayesian) optimization process (26–30). These approaches describe (individual or collective) agent optimal choices as the result of information they obtain in a noisy, but knowable environment, with information dynamics that are fundamentally Bayesian. This connection between optimal intertemporal decisions, information, and fitness (growth) was previously explored as a mathematical formalism to optimize betting and portfolio investment returns (31, 32). However, its applications to human behavior and population dynamics suggest that it serves a suitable basis for the general statistical mechanics of wealth growth and inequality (30).

Here we unify these approaches to develop a statistical dynamics of growth and inequality in a population of strategic agents, where the growth rates result from investing and learning in a stochastic environment.

In this approach, heterogeneous agents invest in sequential, stochastic environmental events based on signals they perceive as they go, and grow their wealth based on the quality of their predicted allocations. By exploring this mechanism of (optimal) information-driven growth in the context of population dynamics, we obtain a better understanding of how wealth growth and disparities originate from differences in agent knowledge and adaptive behavior. More broadly, this work adds a new dimension to the study of wealth inequality that more fundamentally links inequalities between wealth, growth, and agents’ subjective characteristics, such as their present knowledge, their singular life-course experience, and the quality of their knowable environment, e.g. in terms of its opportunities expressed as statistical rates of return on investments.

Our approach treats both resources and information as dynamically coupled quantities. To model information dynamics, we show that learning in the joint space of environmental states and agents’ signals is developed optimally in terms of Bayesian inference, translating a maximization of the predictability of environmental states into that of resource allocations and growth.

We finish by exploring the general consequences of learning a shared environment on the statistics of information and resources, and discuss the consequences for the role of general education and training on population dynamics and its potential to reverse long-term wealth inequality (14).

## Theory and modeling of information-based growth

We start by deriving a general theory of growth rates in terms of informational quantities. Here, information means an agent’s predictive knowledge of event probabilities in a noisy environment. Agents seek to maximize the growth of their resources over time by investing in a set of possible events in their environment using their individual knowledge. Agent’s knowledge is subjective, as it is formed by the agent’s own experience, model of the world, and expectations (“beliefs”), which are assumed here not to be shared or compared with other agents. The agent’s beliefs are adjusted by observing environmental outcomes in time through an iterative process of (Bayesian) learning. After developing the general framework, we illustrate these dynamics using a multinomial model of discrete environmental states and choice, for which we derive closed-form expressions for the average resource growth rate and volatility in terms of information-theoretic quantities. We will then identify the general circumstance when this learning process dynamically attenuates inequality in resource growth rates across populations.

### Growth from information

We consider a population of *i* = 1, …, *N* agents, each with initial resources *r*_*i*_ that can be (re)invested into the set of outcomes of their environment to generate returns. The agents have access to a private signal *s* ∈ *S*, which they use as a predictor to invest resources in events *e* ∈ *E* generated by their environment. The set of signals and events are described by the joint probability distribution, *P*(*E*, *S*), with marginals *P*(*E*) and *P*(*S*).

At every time step, each agent observes its own signal *s* and allocates resources *r* on events following a vector *B*(*E*|*s*), such that $\sum_{e}B(e|s)=1$, *e* ∈ *E*. As the event *e* is revealed, the agent is awarded returns, *w*_*e*_, for the fraction of resources invested in the correct outcome, *B*(*e*|*s*)*r*_*i*_. After *n* steps, the agent’s total resources (wealth) are


(1) $$r_{n}=r_{i}\prod_{\;j=1}^{n}B(e_{j}|s_{j})w_{e_{j}}=r_{i}\prod_{s,e}[B(e|s)w_{e}{]}^{W_{s,e}},$$

where *W*_*s*,*e*_ is the number of occurrences (“wins”) of *s*, *e*. By the law of large numbers, *W*_*s*,*e*_/*n* → *P*(*s*, *e*) as *n* → ∞. It follows that the average growth rate of resources over large *n* steps is


(2) $$\gamma_{i}\equiv \frac{1}{n}\log\;\frac{r_{n}}{r_{i}}\approx \sum_{e,s}P(s,e)\log\;[B(e|s)w_{e}].$$

Kelly showed that the maximal growth rate as *n* → ∞, obtained by maximizing Eq. (2) with relation to *B*(*E*|*S*), results in an allocation mirroring the conditional probability, *B*(*E*|*S*) = *P*(*E*|*S*). This maximum growth rate is the mutual information, *γ*_max_ = *I*(*E*, *S*) when the odds are “fair,” *w*_*e*_ = 1/*P*(*e*) (31).

**Fig. 1.**
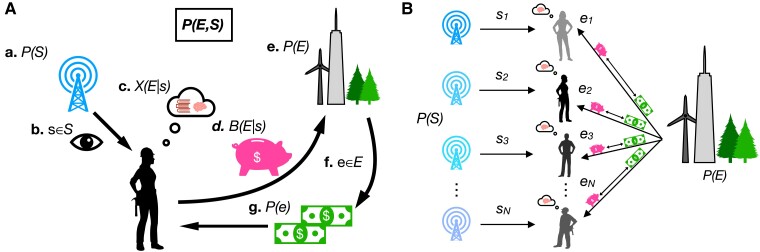
General dynamics of learning and growth: agents obtain resources from their environment based on the quality of their information. A) At each time step, (a) the agent’s private channel (memory, senses) outputs a signal *s* ∈ *S* with probability *P*(*s*). (b) The agent observes the state *s* and (c) consults their belief for the conditional outcome probability of the environment, *X*(*E*|*s*). (d) The agent makes proportional resource allocations on all possible outcomes *B*(*E*|*s*). (f and e). The true event *e* ∈ *E* is revealed from the environment with probability *P*(*e*), and (g) the agent receives a payout proportional to the marginal probability of *e*. B) In a population simulation, *N* agents independently sample private signals and invest in events sampled from the same environment.

Typical agents do not start out with perfect knowledge. In this case, agents must invest resources using their best estimate for the conditional probability, *X*(*E*|*S*) ≠ *P*(*E*|*S*). Then, their resource growth rate will be lower than the maximum. This can still be written in terms of informational quantities as the Kelly growth rate (SM 1),


(3) $$\gamma =I(E;S)-E_{s}(D_{KL}[P(E|s)\|X(E|s)]),$$

where E_*s*_ is an expectation value over the states of the signal, and $D_{KL}[P(E|s)\|X(E|s)]=\sum_{e}P(e|s)\log\;(P(e|s)/X(e|s))\geq 0$ is the Kullback–Leibler divergence, expressing how similar the two distributions in its inputs are. This general result shows that agents with better information will experience greater resource growth rates, as long as they invest optimally (33). These compounding dynamics are illustrated in Fig. 1. We also see that this setup allows us to consider agents with different knowledge, corresponding to skill heterogeneity within a population. We will discuss other general issues of innovation and structural position as we introduce learning in populations below.

We note that, in reality, people may be in debt typically leading to a negative additive component of their growth rate due to interest payments. We do not consider this situation here, except to point out that if such a component is constant it does not affect our analysis. If, however, the loan rate can be reduced via better information, it will add another dimension to the optimization of the overall growth rate.

We will now illustrate these general results using a specific, stationary multinomial model. While the theory is developed for general environmental dynamics, its limitation to a stationary environment will allow us to derive quantities of mean growth rate and volatility, familiar to geometric Brownian motion (GBM) in closed-form and establish the parallels to most wealth-growth models. This model will allow us to then illustrate and simulate the population dynamics of growth and inequality among agents with heterogeneous information. Later, we will also show how agents can improve their information optimally over time through a process of iterative Bayesian learning.

#### Multinomial choice model

Consider the space of signals *S* and environmental states *E* of equal size *l*, with outcomes *s*, *e* ∈ 1, …, *l* and degenerate multinomial conditional probability


(4) $$P(e|s)=f(p,l)=\left\{ \begin{array}{cc} p & \text{if }s=e,\\ \frac{1-p}{l-1} & \text{if }s\neq e, \end{array}\right.$$

where 0 < *p* < 1 is the binomial probability of guessing the correct environmental outcome. For simplicity, we assumed that the probability of a correct guess is independent of *l*. The distribution has uniform marginals, *P*(*e*) = 1/*l* and *P*(*s*) = 1/*l*, for all signals and events, such that *P*(*s*|*e*) = *P*(*e*|*s*) via Bayes’ rule.

With these choices, we can derive expressions for the relevant informational quantities in closed-form. The mutual information between an agent’s signals and environmental outcomes is then *I*(*E*; *S*) = log *l* + *p*log *p* + (1 − *p*)log ((1 − *p*)/(*l* − 1)) (Appendix Eq. 4). As the simplest illustration, for a binary choice, *l* = 2, the first term gives 1 bit of entropy of the environment and the remaining terms give the conditional entropy, expressing how well an agent could know the environment given their signal. In the limit *p* → 1, the signal gives agents perfect knowledge of *P*(*E*).

So far we considered that the agent has perfect knowledge of the joint distribution of the signals and the environment. When this is not the case, we can write a parametric expression of the agent’s ignorance in terms of an estimated binomial probability *x* ≠ *p*. The agent’s likelihood model of the conditional probability is then *X*(*e*|*s*) = *f*(*x*, *l*). The divergence term of Eq. 3 becomes the divergence between *f*(*p*, *l*) and *f*(*x*, *l*) averaged over all signals, E_*s*_[*D*_*KL*_] = *p*log (*p*/*x*) + (1 − *p*)log ((1 − *p*)/(1 − *x*)). Subtracting the mutual information by this term yields the agent’s actual growth rate under imperfect information as (supplementary material, Appendix B)


(5) $$\gamma =\log\;l+p\log\;x+(1-p)\log\;\frac{1-x}{l-1}.$$

**Fig. 2.**
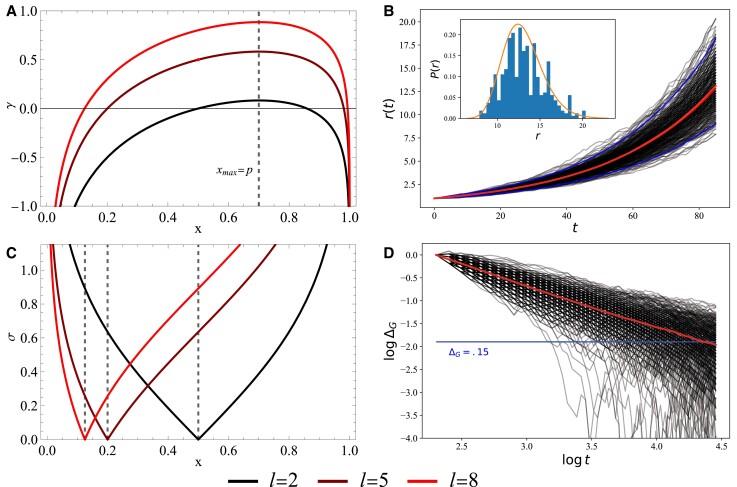
Example of parameters and dynamics for wealth growth without learning. The growth rate and volatility are computed analytically for a discrete multinomial environment, reproducing the limit of GBM dynamics. A) For *p* = 0.7, the growth rate maximizes at *x* = 0.7, decreases as *x* diverges from *p*, and scales with *l*. The parameter *l* = 2 provides a realistic range of average growth rates. B) Monte-Carlo simulations with *N* = 388 homogeneous agents, all with *γ*(*x*) = 0.03 and *r*_0_ = 1. The 95% confidence interval is plotted, as are the overlapping expected mean, predicted by *γ* = 0.03, and the population sample mean. *Inset*: The resource histogram is fit to a log-normal distribution of the same growth and volatility parameters. C) Volatility is minimized at *x* = *1/l* and increases monotonically in either direction. Volatility increases more rapidly at higher values of *l*. D) Over time, $\Delta_{\gamma }\to 0$ as agents’ growth rates approach the Kelly growth rate. The average agent converges to within 15% the expected mean at *t* ≈ 80.

This expression is plotted in Fig. 2A as a function of *x* for various *l* values and fixed *p*. We see that increasing the size of the event space, *l*, reduces the probability of any individual outcome, increasing the payouts and the Kelly growth rate. The maximal growth rate is obtained when *E*_*e*_ [*D*_*KL*_] → 0, when *x* → *p*. Conversely, *γ* → 0 when *p* → 1/*l*, indicating the signal and the environment have become statistically independent.

Treating *γ* as the expected resource growth rate, the volatility is calculated as the second moment of the growth process. The volatility squared (variance) is given as (Appendix C)


(6) $$\sigma^{2}=\;p(1-p)\log^{2}\;\frac{x(l-1)}{1-x}.$$

This expression is shown in Fig. 2C. The volatility vanishes in the limit *x* → 1/*l*, corresponding to when agents invest indiscriminately with equal probability in all possible event types. A larger *l* increases the magnitude of the growth rate, but also the volatility. The volatility is highest when *p* → 1/2 and the environment is most uncertain. In any case, the agents feel surest of the outcomes when *x* → 0 or *x* → 1.

Kelly’s formulation describes the average growth rate of resources over a large number of discrete investments (31). To derive a growth process in time, we average over *ω* bets per unit time, such that Δ*t* = 1/*ω* is the interval of time between investment periods. Returns at time *t* + Δ*t* are then the mean of all investment returns earned in the time interval [*t*, *t* + Δ*t*]. In the limit *ω* → ∞, as the agent makes continuous allocations, *r*_*n*_ → *r*(*t*) and *γ* describe the average growth rate. We consider *t* ≈ 10^−2^year (i.e. 1% a year) so that our simulated results are comparable to previous work based on yearly growth rates of the order of a few percent. Volatility is reduced $\sigma_{t}=\sigma_{n}/\sqrt{\omega }$ as fluctuations are averaged out in each time step (supplementary material 10).

Fig. 2C demonstrates the two investment regimes for each value of *γ*, where the growth rate maps to either high or low volatility depending on the value of *x*. Investments with *x* > *p*, which we describe as *aggressive*, overestimate the dependence between the signal and environment. Under this condition, agents invest relatively more on diagonal outcomes and experience large gains or losses resulting in higher volatility. With *x* < *p*, which we denote *conservative*, agents underestimate *p* and distribute their wealth more equally across all outcomes, resulting in less volatility. Agents can also experience *γ* = 0 at two values of *x*: In the trivial limit, as *x* → 1/*l*, signals and agent investments become statistically independent. The other trivial case can be solved for numerically when *γ* = 0.

With given *x* independent of time, the dynamics reduce to the well-known behavior of GBM with drift. Fig. 2B shows the dynamics of a population of agents with homogeneous (non-time dependent) parameters evolved using a Monte-Carlo simulation. In this particular situation, mean population resources grow with $\left\langle r(t)\rangle =(1/N)\sum_{i}r_{i}(t)=\exp\;[\gamma t\right]$, in agreement with (12).

We also demonstrate that the time-averaged growth rate of resources converges to the Kelly growth rate over many allocations. Fig. 2D shows the asymptotic convergence of the normalized difference of averaged growth rate for individual agents Δ_*G*_ = (*γ* − *G*)/*γ* → 0, where *G* = (1/*t*)ln(*r*(*t*)/*r*(0)) (dark) and population-averaged growth rate $\langle G\rangle =(1/N)\sum_{i}G_{i}$ (light).

We have thus far considered *x* as a static variable and explored the dynamics of resources when *x* ≠ *p* in a stationary environment. To converge to maximal growth rates, however, it is necessary that agents can estimate the correct event properties, given their signals, a situation to which we now turn.

### Dynamical growth rates from Bayesian inference

Realistic agent trajectories are dynamical, reflecting investment allocations that are history-dependent and result from the cumulative knowledge of each agent’s past experience (12, 34). Agents must then improve their information about the environment by updating their model of the conditional relationship of *S*|*E* with each observation. In the absence of other random processes, this learning task is optimally achieved in terms of sequential Bayesian inference (35, 36):


(7) $$X_{n}(e|s)=AP(s_{n}|e_{n})X(e_{n})=\left[\Pi_{i=1}^{n}\frac{P(s_{i}|e_{i})}{P(s_{i})}\right]X(e),$$

where the normalization $A=(\int de_{n}P(s_{n}|e_{n})X(e_{n}){)}^{-1}$. We also take the prior probability, *X*(*e*_1_) = *X*(*e*), because we are assuming that the environment is stationary or at least slowly changing relative to agents’ learning rates.

**Fig. 3.**
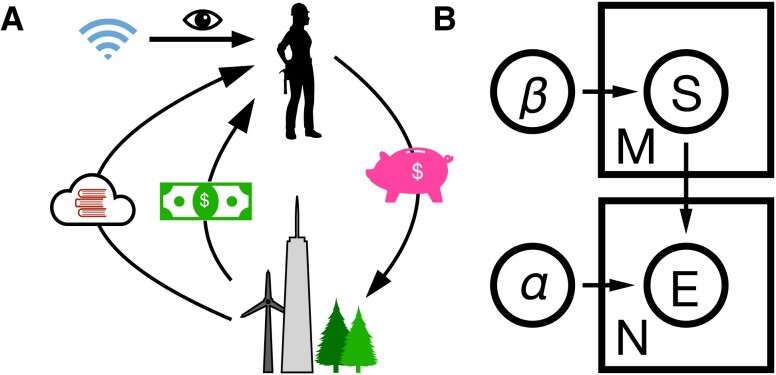
Schematic illustration of the learning process. A) In addition to earning resources, the agent obtains information with each investment in the environment. B) Notation for the latent Dirichlet inference process. The agent is assigned prior parameters α,β, corresponding to their belief for the distributions of *E* and *S*, which are updated based on event counts *M*, *n* respectively.

Then, Bayesian inference converges *X*(*E*|*S*) → *P*(*E*|*S*), decreasing the information divergence over long times. Through interactions with the environment, the agent optimally gathers information (30) as well as resources as demonstrated in Fig. 3A. Specifically, by minimizing the information divergence, learning agents maximize their resource growth over the long term.

This formalism allows us to start considering general aspects of innovation in heterogeneous populations, including issues of competitive advantage and structural positions in terms of agents’ initial knowledge, models of the environment, and embedding within socioeconomic networks. Regardless of any of these elaborations, any learning model aspiring to optimal prediction must be Bayesian, as it is the single best way to incorporate observed data towards making predictions of future states of the environment (37) and maximizing long-term growth.

There is growing interest in incorporating learning agents in economics and other social sciences towards formulating models of more realistic “rational expectations” in intertemporal optimization problems (38). At present, however, most of these approaches adopt simplified learning models, for example, based on least-squares minimization (38), which at best apply in particular cases, such as for Gaussian likelihoods. Consequently, we see a wide range of interesting opportunities in the social sciences for the adoption of more explicitly Bayesian frameworks, as has become increasingly common in psychology (39).

In the following subsection, we describe a parametric Bayesian inference scheme applied to the multinomial model via a Dirichlet prescription of conjugate priors (40), before we return to the general case to discuss issues of inequality in the light of learning.

#### Bayesian dynamical growth in the multinomial model

To illustrate these learning dynamics, we now return to the multinomial model of choice. We define the agent’s likelihood function of a sample of the signal, *s*|*e*, as a categorical distribution with parameter vector $\beta =\{\beta^{1},\ldots ,\beta^{l}\}\in R^{l}$, with each vector corresponding to an event and each component, $\beta_{s}^{e}$, corresponding to a signal, event pair. The probability mass function is given by $P(s|e)=\prod_{s}(\beta_{s}^{e}{)}^{s}$, with normalization $\sum_{s}\beta_{s}^{e}=1$. The conjugate prior distribution of *E* is given by a Dirichlet with hyperprior vector $\alpha \in R^{l}$, and distribution *P*(*e*) = *α*_*e*_/*A*, where magnitude $A=\sum_{e}^{l}\alpha_{e}/l$. This scheme is illustrated in Fig. 3B.

We set *α*_*e*_ = 1 for all *e* so that our prior is uniform, for simplicity. We ensure the off-diagonal degenerate condition by setting $\beta_{s}^{e}=p_{e}$ for *s* = *e*, and for off-diagonal events, *e* ≠ *s*, $\beta_{s}^{e}=\left(1-p_{e}\right)/\left(l-1\right)$, satisfying Appendix Eq. 2. The binomial parameter describing the environment is then given by the average along the diagonal


(8) $$p=\frac{1}{l}\sum_{s}^{l}\beta_{s}^{e},\;s=e.$$

An agent with imperfect information will have estimates for the parameters, $\tilde{\alpha }\neq \alpha$ and $\tilde{\beta }\neq \beta$, and posterior, $X(E|S,\tilde{\beta },\tilde{\alpha })\neq P(E|S,\beta ,\alpha )$. With each observation, the agent must update *X*(*E*|*S*) via (Appendix E)


(9) $$X(e|s)\propto \frac{m_{\left(-s\right)}^{\left(-e\right)}/\omega k+{\tilde{\beta }}_{s}^{e}}{M^{\left(-s\right)}/\omega k+1}(n_{\left(-e\right)}+{\tilde{\alpha }}_{e}),$$

where $m_{\left(-s\right)}^{\left(-e\right)}$ and *n*_(−*e*)_ are the total numbers of samples *e*, *s* and *e* excluding the current, and $M^{\left(-s\right)}=\sum_{e}m_{\left(-s\right)}^{\left(-e\right)}$ is the number of samples of *s* excluding the current. We also introduce an inference time, *k*, as a free parameter that weighs the evidence versus the prior, with units *time*/*update* such that *t*/*k* is unit-less. In the limit *k* → ∞, the agent does not update their prior with new evidence. In the opposite limit, *k* → 0, the agent ignores the prior and considers only the most recent evidence, and this becomes a maximum likelihood model.

During the inference process, the agent will break the degeneracy of their posterior as they infer each $\beta_{s}^{e}$ individually. This is inconsequential though, as *x*(*t*) can still be computed similarly to Eq. 8 at any time. The degeneracy of *P*(*E*|*S*) permits us to reduce the dynamics of *X*(*E*|*S*) to that of the diagonal probability *x*(*t*), such that (Appendix F)


(10) $$x(t)=\frac{\;pt/kl+x_{0}}{1+t/kl},$$

where *x*_0_ is the agent’s initial binomial probability parameter. This equation illustrates the core results of this approach, as the dynamics of the information of the agent’s posterior determine the average dynamics of the growth rate via the functional, *γ*[*x*(*t*)]. Over many observations, the agent optimizes their guess, driving *X* → *P*, minimizing their information divergence as *D*_*KL*_(*P*||*X*) → 0. The agent thus maximizes the average growth rate for their signal over time with a power law −1 in terms of the dimensionless inference parameter *λ* ≡ *t*/*kl*, at larger times *λ* ≪ 1. As previously mentioned, though, agents who have maximized information are still subject to the volatility of sample fluctuations. For the remainder of this paper, we will study the effects of this learning process on the population dynamics of growth rates and wealth.

## Population effects of information dynamics

Having defined the dynamics of information and resources for single agents, we can now explore the general dynamics of growth rate statistics in a heterogeneous population and its implications for long-term inequality. Mean growth rates can vary because of a number of different factors. Particularly, agents have different initial conditions of knowledge, they experience different environmental stochastic histories, and they may have different models of the world in terms of their likelihood functions. We will now explore these sources of information heterogeneity and show that with a shared statistical signal, a population can reverse the (dominant) effects of heterogeneity on growth and inequality (14).

We write the population variance of growth rates generally in terms of information-theoretic quantities, where *I*_*i*_ ≡ *I*(*E*; *S*_*i*_) and $D_{i}\equiv E_{s_{i}}(D_{KL}[P(E|s_{i})\|X(E|s_{i})])$. The population variance is given as (Appendix G)


(11) $${\mathrm{Var}}_{N}[\gamma_{i}]={\mathrm{Var}}_{N}[I_{i}]+{\mathrm{Var}}_{N}[D_{i}]-2{\mathrm{Covar}}_{N}[I_{i}D_{i}].$$

The first term is independent of any agent’s imperfect knowledge or learning process and depends only on their model (likelihood) of the environment, given the agents’ signals.

The second term expresses variance in the prior and different learning trajectories across agents. This term vanishes as agents learn their environment fully. It follows that these two sources of variance vanish only if every agent has the same model in a shared environment with the same statistics, and after every agent has had time to learn their environment.

These two terms also express formal distinctions between the familiar Keynesian formulation of intrinsic uncertainty versus risk in socioeconomic behavior. Agents cannot know a priori what type of uncertainty they are facing and must learn as best as they can from their experience. A misspecification of the agents’ model of the world, via an incorrect likelihood function, will result in irreducible uncertainty and a lower growth rate than possible. In terms of communications theory, this situation effectively uses the environmental experience suboptimally, by picking a signal that does not maximize the channel capacity, as the largest possible mutual information between the agents’ signal and events in the world (32). On the other hand, risk in the sense of probabilistic events with a known distribution can be reduced (and better assessed) via the Bayesian inference process which builds the correct risk model within a family of functions, by learning its parameters.

The third term is less familiar and arises in populations where the magnitude of the agents’ information co-varies with agents’ divergence from the environment. This may happen in reality when different (likelihood) models of the world co-exist in a population of agents, and when, in addition, less experienced agents with shorter learning histories adopt preferentially some of these models. For example, a younger generation may have a better model of the world but less experience, creating a negative covariance. Or a positive covariance may be generated if learners with a better model are encouraged to learn faster, and others discouraged, creating a kind of cumulative advantage in terms of better information and faster learning. Such situations may provide principled modeling strategies to better understand the success of a posteriori exceptionally successful individuals, and identify situations of competitive advantage in access to information and learning.

### Population effects in the multinomial model

We now illustrate how the inference dynamics happen in the context of the multinomial model. We focus on agents with identically distributed signals, i.e. with the same likelihood function and a shared environment, expressed by the second term in Eq. 11. Thus, we (implicitly) take Var_*N*_[*I*_*i*_] = 0, thereby also eliminating the third term. This situation models a homogeneous population in terms of models of the world, such as for individuals of the same species in a common habitat, or workers in the same industry, with similar training. We will return to the more general case and discuss future opportunities in the discussion at the end of the paper.

**Fig. 4.**
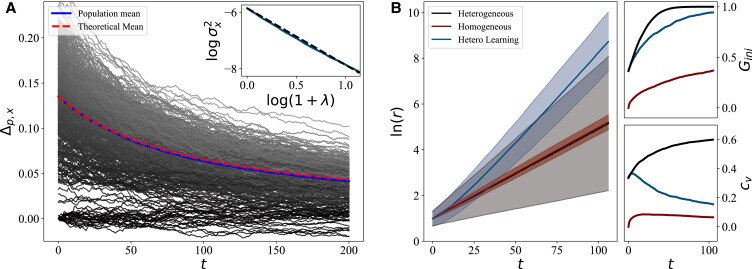
Monte-Carlo simulations of a population undergoing wealth dynamics and Bayesian learning with parameters of mean growth rate, $\bar{\gamma }=0.04$, and standard deviation, $\sigma_{\gamma }=0.641\bar{\gamma }$. A) The simulated and theoretical means of *x* converge to *p*, thus maximizing growth rates. The parametric variance, $\sigma_{x}^{2}$, (dashed) follows the theoretical prediction (solid). The linear behavior log–log plot demonstrates the power law behavior of $\sigma_{x}^{2}$. B) The mean resources of three population types are plotted with a shaded region providing 95% confidence interval bounds for single agent trajectories. Heterogeneity broadens the range of possible wealth values while learning increases mean growth while narrowing the shaded region relative to no inference. Agent learning slows the increase in the Gini coefficient introduced by heterogeneity and reduces the coefficient of variation.

For a population of agents independently sampling a shared multinomial environment, the initial variance in growth rates is given by the variance in the initial binomial parameter, $\sigma_{x}^{2}$. The dynamics of the variance in binomial parameter for a population of size *N* is (SM 22)


(12) $${\mathrm{Var}}_{N}[x_{i}(t)]\equiv \langle [x_{i}(t)-\langle x(t)\rangle {]}^{2}\rangle =\frac{\sigma_{x}^{2}}{(1+t/kl{)}^{2}},$$

where $\left\langle x(t)\rangle =(1/N)\sum_{i}x_{i}(t\right)$. Assuming a population of entirely conservative (or aggressive) agents, such that all growth rates map to a unique binomial parameter, we can approximate the variance in growth rates, $\sigma_{\gamma }^{2}(t)=\langle (\gamma [x_{i}(t)]-\gamma [\langle x(t)\rangle ]{)}^{2}\rangle$, by Taylor expanding the second moment of the resource distribution. The approximation carried out in SM 38 shows that the growth rate variance decreases asymptotically in polynomial *t*^−2^ time (41). Fig. 4A demonstrates that in a population of agents sampled from a Gaussian distribution of growth rates and resources learning their environment, Δ_*p*,*x*_ = *p* − *x*(*t*) → 0 as *t* → ∞, and individual binomial parameters converge to the optimal value. At the population level, there is an agreement between the empirical population mean and theoretical mean trajectory, calculated by evolving 〈*x*(*t*)〉 using Eq. 10. Similarly, the empirical population variance in *x* matches the theoretical power law prediction given by Eq. 12.

These results show that learning a shared, stationary environment reduces growth rate variance on the same time scale as the dynamical effects introduced by growth rate variance (14). This shows that fast learning (sufficiently low *k*) equalizes information access and is a suitable mechanism for reversing the long-term effects of heterogeneous growth on inequality.

We demonstrate these features of the dynamics by comparing the statistics of resources across Monte Carlo simulated populations. We first use homogeneous initial conditions, then heterogeneous initial conditions with and without inference. To measure the increase in inequality, we track the Gini coefficient, denoted *G*_ini_, which varies between zero—for uniformly distributed resources—and 1, for maximally unequal wealth distributions (For a lognormal distribution, such as in the GBM model, $G_{\mathrm{ini}}(t)\approx \mathrm{Erf}[\sigma_{r}^{2}(t)]$.) Additionally, we measure the relative increase in standard variation to the mean of resources via the coefficient of variation, *c*_*v*_ = *σ*_*r*_/〈*r*〉. More on this analysis is given in Ref. (14).

The resource time evolution shown in Fig. 4B demonstrates that growth rate heterogeneity dramatically broadens the wealth distribution, in agreement with (14). Accordingly, heterogeneity increases *G*_ini_ and *c*_*v*_ as compared to a homogeneous population. The introduction of learning increases the average growth rate in a heterogeneous population, as demonstrated by the higher mean wealth, while reducing the variance in resources. The former slows the rapid increase *G*_ini_, while the combination of both reduces *c*_*v*_ to levels comparable to the homogeneous trajectory, confirming that learning reverses the effects of heterogeneity on inequality.

While this simplified model does not capture the nuanced effects of educational systems or skill heterogeneities implied in real societies, the connection between convergent learning in a population and growth is general and provides a sound theoretical basis for the observed benefit of education on national growth, human capital, and inequality reduction (42–44).

## Conclusion

In this paper, we developed a statistical dynamical theory for the origin of resource growth rates in populations of learning agents experiencing a shared stochastic environment. We showed that an agent’s growth rate is, in the limit of many decisions, the quantity of mutual information between their signal and the environment and that learning through Bayesian inference provides a natural and necessary (optimal) mechanism for increasing agents’ growth rates, managing volatility, and reducing growth disparities across populations over time. We demonstrated that in the particular static case (without learning), this framework re-produces GBM models widely used in wealth dynamics and inequality studies and provides models for their parameters. When agents can learn, their parameters become optimal over time and acquire formal interpretations in terms of information.

The present treatment answers an important open question on how to mechanistically control variances in growth rates across a society while maximizing learning and growth and generally enriches the typical modeling schema of wealth dynamics by incorporating agents’ subjective choices in a structured, stochastic but knowable environment. This work also adds to the foundations necessary for incorporating formal models of information and strategic subjective agent behavior in statistical mechanics, helping bridge a gap between physics and computer science, and biological and social sciences.

There are a number of interesting developments that this theoretical framework suggests for modeling more realistic, particular situations. First, learning is never quite uniform across populations or time, varying across the life course, with some agents being able to dedicate more time and effort to it than others. This issue can be modeled by making inference rates dynamic and heterogeneous, for example, through coupling to agents’ socioeconomic status (SES) or age. Importantly, lower SES has been shown to be correlated with the presence of stressors that inhibit the cognitive ability of people to learn (45–47), while higher SES correlates with better educational outcomes (48–50). Coupling learning rates with SES would alter the population’s learning trajectory and potentially attenuate its effectiveness in reducing information and wealth inequality. Moreover, our analysis has assumed that each agent samples identically distributed signals. In reality, people across different structural positions in social networks, for example, associate with place, gender, or race/ethnicity, typically have differential access to signals (opportunities), with implications for what they can learn and for resulting social equity. Along with different signals, different agents may have different models of the world, which are naturally incorporated in the scheme developed here by different likelihood functions in Bayesian learning. We have shown in general how such heterogeneities among agents will result in inequalities in their growth rates, but many interesting situations remain to be explored in the future. Finally, real societies also feature interactions between agents and planners that redistribute wealth, economic rents, and heterogeneous frictions that further shape wealth dynamics. The work developed here emphasizes the importance of understanding these policy choices and socioeconomic phenomena through the lens of how they affect specific wealth dynamical parameters, namely initial wealth statistics versus growth, including average growth rates and volatilities across populations. Future studies of the origins of inequality and social equity should consider these structural complexities from the general point of view of access to information and learning, and the specific analytical tools that they introduce.

Second, from the point of view of maximizing future resources, there are familiar trade-offs between learning and investing. These can be modeled in terms of the inference process divided into passive experiential learning, resembling the “learning by doing” featured above, and, additionally, emulating formal, institutional education wherein agents sacrifice short-term wages to more rapidly acquire information. These considerations define agent trade-offs between actively exploring and passively exploiting the environment, an important research topic in both experimental neuroscience and machine learning (51, 52). Furthermore, while information is a non-rival quantity that can be made available to a society with minimal cost of sharing or degradation from use, the generation and dissemination of information through teaching is a costly process that can produce additional non-trivial dynamics. Agents must also consider the cost and benefits of seeking education in non-stationary environments, where the value of information may fluctuate or decay over time. Expressing the social costs of education through mechanisms of finite learning resources could help explore trade-offs in investing in human capital over various timescales of learning and environmental evolution (53, 54) and help determine when they are worth it—for individual agents and societies—in intertemporal settings.

Third, tracking individual agent dynamics under constraints of finite (varying) lifespans can help determine the effects of generational wealth transfers on inequality, and provide insight into life-course strategies (55) and issues of valuing (and discounting) the future. Thus, an extended framework can help us explore the scope of education under the discounting of delayed resources by longevity and lived volatility (56); including the implications of costs and expected earnings with or without an education over time. Lastly, agents in this model experience the same environment and learn the same information, whereas actual communities specialize in different, complementary skills that may minimize knowledge redundancy. These information complementarities and exchanges are known commonly in the social and ecological sciences in terms of the division of labor and knowledge (57). How agents decide which information to learn and what profession to choose based on their environments begets different growth rates across a population, altering emerging inequality and influencing how social groups cooperate or compete across community or institutional social levels (58). Cooperation among agents with synergistic information in a stochastic environment has been shown to produce non-linear additive effects on aggregate information (59), suggesting that cooperative agents would experience larger growth rates when coordinated, compared to the sum of agents acting independently (60, 61). Studying this connection between social behavior and growth from the point of view of information and learning will provide insights into the circumstances when cooperative and altruistic behavior becomes favored both via sharing resources and information.

## Supplementary Material

pgad093_Supplementary_Data

## Data Availability

The data underlying this article are available with DOI/accession number(s): 10.5281/zenodo.7114564.

## Preprints

A preprint of this article is available online at https://doi.org/10.48550/arXiv.2209.09492
