# Supplementary material for: Learning increases growth and reduces inequality in shared noisy environments
Source: PNAS Nexus. 2023 Mar 22;2(4):pgad093. doi: 10.1093/pnasnexus/pgad093 (PMC10109450; doi:10.1093/pnasnexus/pgad093)
Supplement: pgad093_Supplementary_Data [file pgad093_supplementary_data.zip › PNASNexus2022_InformationGrowth.pdf]

# Supplementary Materials for: Learning Increases Growth and Reduces Inequality in Shared Noisy Environments


Jordan T. Kemp[1] and Luís M. A. Bettencourt[2,3]

[1]Department of Physics, University of Chicago, Chicago, Illinois 60637, USA
[2]Department of Ecology and Evolution, University of Chicago, Chicago, Illinois 60637, USA and
[3]Mansueto Institute for Urban Innovation, University of Chicago, Chicago, Illinois 60637, USA



(Dated: June 5, 2024)


## I. SUPPLEMENTARY MATERIAL

### A. Information quantities

#### 1. Kelly growth rate

Multiplying and dividing by $P(e|s)$ in the logarithm of Eq. 2 yields

$$\begin{aligned}\gamma &= \sum_{e,s} P(e,s)\log\left[w_e P(e|s)\frac{X(e|s)}{P(e|s)}\right]\\ &= \sum_{e,s} P(e,s)\log\frac{P(e|s)}{P(e)} - P(s)P(e|s)\log\frac{P(e|s)}{X(e|s)} \\ &= I(E;S) - \mathrm{E}_s\big(D_{KL}\big[P(E|s)||X(E|s)\big]\big),\end{aligned} \tag{1}$$

where $\mathrm{E}_s$ is an expectation value over all signal states.

### B. Simplified growth model

Consider a conditional probability that is degenerate off-diagonal,

$$P(e|s) = f(p,l) = \begin{cases} p & \text{if } s = e, \\ \frac{1-p}{l-1} & \text{if } s \neq e. \end{cases} \tag{2}$$

The "correct" outcome corresponding to the sampled event occurs with conditional probability $0 < p \leq 1$, and all other "incorrect" guesses occur with some uniform probability normalized to

$$\sum_{e}^{l-1} P(e|s) = 1 - p; \quad s \neq e. \tag{3}$$

We describe the agent's posterior for all agents with the same form, with the "correct" binomial coefficient $x$. Thus, we calculate the growth rate by taking the expectation value of the posterior over the set of signals, summing over diagonal and off-diagonal components separately.

The mutual information separates into a term of only $l = 1/P(e)$, an on-diagonal, and off-diagonal term

$$\begin{aligned} I(E;S) &= \sum_{e,s}^{l} P(e,s)\big[\log l + \log P(e|s)\big] \\ &= \log l + p\log p + (1-p)\log\frac{1-p}{l-1} \\ &= H(E) - H(E|S),\end{aligned} \tag{4}$$

with the entropy of the outcome given by $H(E) = \log l$ and the reduction in entropy by the signal given by $H(E|S) = -p\log p - (1-p)\log\frac{1-p}{l-1}$. The information maximizes as $p \to 1$ and increases with $l$, and vanishes at $p \to 1/l$. The divergence is

$$\begin{aligned} \mathrm{E}_s\big[D_{KL}(P||X)\big] &= \sum_{e,s} P(e,s)\log\frac{P(e|s)}{X(e|s)} \\ &= p\log\frac{p}{x} + (1-p)\log\frac{1-p}{1-x},\end{aligned} \tag{5}$$

which is always non-negative and vanishes when $x \to p$. We can write the growth rate as the difference between these two terms as

$$\gamma = \mathrm{E}\big[\log lf(x,l)\big] = \log l + p\log x + (1-p)\log\frac{1-x}{l-1}. \tag{6}$$

### C. Variance of growth model

The volatility can be calculated via the second moment of the stochastic growth rate as

$$\sigma = \sqrt{E\big[\log\big(lf(x,l)\big)^2\big] - E\big[\log lf(x,l)\big]^2}. \tag{7}$$

$\mathrm{E}\big[\log lf(x,l)\big]$ is simply $\gamma$, and the second term is

$$\begin{aligned} \mathrm{E}\big[\log lf(x,l)\big]^2 &= \left(\log l + p\log x + (1-p)\log\frac{1-x}{l-1}\right)^2 \\ &= \log^2 l + p^2\log^2 x + (1-p)^2\log^2\frac{1-x}{l-1} \\ &\quad + 2p\log l\log x + 2(1-p)\log l\log\frac{1-x}{l-1} \\ &\quad + 2p(1-p)\log x\log\frac{1-x}{l-1}.\end{aligned} \tag{8}$$

The first term expands to

$$\begin{aligned}\mathrm{E}\big[\log\big(lf(x,l)\big)^2\big] &= \mathrm{E}_{e,s}\big[(\log P(e|s)+\log l)^2\big]\\ &= \log^2 l + p\log^2 x + (1-p)\log^2\frac{1-x}{l-1}\\ &+ 2p\log l\log x + 2(1-p)\log l\log\frac{1-x}{l-1}.\end{aligned} \tag{9}$$

Combining these two quantities yields the volatility, where $(1-p)-(1-p)^2 = p(1-p)$,

$$\begin{aligned}\sigma_n &= \sqrt{p(1-p)\left[\log^2 x + \log^2\frac{1-x}{l-1} - 2\log x\log\frac{1-x}{l-1}\right]}\\ &= \sqrt{p(1-p)}\log\frac{x(l-1)}{1-x}.\end{aligned} \tag{10}$$

The variance of investment clusters of size $1/\omega$ scales as

$$\sigma_t^2 = \frac{1}{\gamma}\sigma_n^2, \tag{11}$$

where the subscript $t$ denotes the temporal variance.

### D. Latent Dirichlet Allocation

In this section, we derive the Latent Dirichlet Allocation (LDA) mode for the degenerate multinomial environment. The Bayesian update equation is given by

$$X(e|s) \propto \frac{(m_{(-s)}^{(-e)} + \tilde{\beta}_s^e)}{(M^{(-s)} + \tilde{B}^s)}(n_{(-e)} + \tilde{\alpha}_e), \tag{12}$$

for $m_{(-s)}^{(-e)}$ number of samples of outcome $s$ conditional on $e$ excluding the current, $n_{(-e)}$ the number of samples of $e$ excluding the current in a batch of $n = \sum_e n_{(-e)}$ trials, where $M^{(-s)} = \sum_e m_{(-s)}^{(-e)}$. We set $\alpha_e = 1$, as every event is equally likely. For $s = e$, $\tilde{\beta}_s^e = x_e$, and for $s \neq e$, $\beta_{es} = \frac{(l-1)}{1-x}$ to impose degenerate off-diagonal conditions on $s|e$. We introduce $\tilde{B}^s = \sum_e \tilde{\beta}_s^e$, whereby symmetry, $\tilde{B}^s \equiv \tilde{B} = 1$, and we count over the diagonals, $n_{(e=s)}$, and off diagonals, $n_{(e\neq s)}$. Therefore the diagonal component of the posterior (approximating the environment) behaves as

$$X(e|s) \propto \frac{\big(m_{(-s=e)}^{(-e)} + x_e\big)}{\big(M^{(-s)} + 1\big)}(n_{(-e)} + 1), \tag{13}$$

and the off-diagonal is

$$X(e|s) \propto \frac{\big(m_{(-s\neq e)}^{(-e)} + \frac{1-x_e}{l-1}\big)}{\big(M^{(-s)} + 1\big)}(n_{(-e)} + 1). \tag{14}$$

### E. Asymptotic, temporal behavior

We introduce the temporal behavior, with two constants. We multiply the number of observations by the observation rate $\omega$, with units *samples/time* and the inference rate $k$, with unit *time/update*. The inference rate counts the number of samples per Bayesian update, and the observation rate counts the updates per unit time. We introduce the inference time, $k$, a hyperprior magnitude that weighs the evidence versus the prior, leaving

$$X(e|s) \propto \frac{(m_{(-s)}^{(-e)}/\omega + \tilde{\beta}_s^e k)}{(M^{(-s)}/\omega + k)}(n_{(-e)}/\omega + k). \tag{15}$$

Over many observations, the law of large numbers argues that each outcome count converges to the environmental posterior with some noise, $\xi_i$ as

$$\begin{aligned}M^{(-s)}/\omega &\to P(s)Nt + \xi_s\\ n_{(-e)}/\omega &\to P(e)Nt + \xi_e\\ m_{(-s)}^{(-e)}/\omega &\to P(s|e)Nt + \xi_{s|e},\end{aligned} \tag{16}$$

where the $\xi$'s are fluctuation terms representing deviations from the mean. Over many i.i.d observations of events, $\xi \to 0$. The marginal terms converge to uniform over all states, and the agent posterior converges to the dynamical distribution

$$X(e,\lambda|s) = \frac{P(s|e)\lambda + X(s|e)}{1+\lambda}, \tag{17}$$

where we have converted to the time domain $t = N/\omega$, and substituted the dimensionless inference sample size $\lambda = t/kl$. Over long times, the distribution converges to the environmental posterior by

$$\begin{aligned}X(e,\lambda|s) &\propto \frac{P(s|e)\lambda + X(s|e)}{P(s)\lambda + 1}\big(P(e)\lambda + \alpha_e\big)\\ &\to \left(P(s|e) + \frac{X(s|e,0)}{\lambda}\right)\frac{P(e)}{P(s)} = P(e|s),\end{aligned} \tag{18}$$

yielding power law time-averaged behavior. At early times, as $t \to 0$ the posterior is proportional to the agent's initial agent posterior, $X(E|S)$, and converges to $P(E|S)$ as $kl \ll t \to \infty$. If agents are initialized with the same diagonal posterior value such that $X(s|e) = X(e', s')$ for all $e = e, s' = s'$, we can assume that the diagonals of an agent uniformly converge to $p$ in time such that $X(s|e) \propto x(t)$ for all $s = e$,

### F. Growth rate population variance

The mean growth rate is computed, where for brevity, the expected divergence for agent $i$ with signals $s_i \in S_i$

is given as $\mathrm{E}_{s_i}\big(D_{KL}\big[P(E|s_i)||X(E|s_i\big]\big) \equiv D_i$, and the mutual information between individual signals and the environment, $I(E;S_i) \equiv I_i$

$$\begin{aligned}\langle \gamma_i \rangle &= \frac{1}{N}\sum_i I(E;S_i) - \mathrm{E}_{s_i}\big(D_{KL}\big[P(E|s_i)||X(E|s_i]\big) \\ &= \big\langle I_i \big\rangle - \langle D_i \rangle,\end{aligned} \tag{19}$$

where angle brackets denote population arithmetic means. The variance in growth rates is calculated

$$\begin{aligned}\mathrm{Var}_N[\gamma_i] &= \big\langle (\gamma_i - \langle \gamma_i \rangle)^2 \big\rangle, \\ &= \big\langle \gamma_i^2 + \langle \gamma_i \rangle^2 - 2\gamma_i \langle \gamma_i \rangle \big\rangle \\ &= \langle I_i^2 \rangle - \langle I_i \rangle^2 + \langle D_i^2 \rangle - \langle D_i \rangle^2 \\ &- 2\big(\langle I_i D_i \rangle - \langle I_i \rangle \langle D_i \rangle\big) \\ &= \mathrm{Var}_N[I_i] + \mathrm{Var}_N[D_i] - 2\mathrm{Covar}_N\big[I_i D_i\big].\end{aligned} \tag{20}$$

When all agents are exposed to the same environment and share the same likelihood, the first and third terms vanish, leaving

$$\mathrm{Var}_N[\gamma_i] = \mathrm{Var}_N\Bigg[\mathrm{E}_{s_i}\Bigg(D_{KL}\big[P(S|s_i)||X(E|s_i)\big]\Bigg)\Bigg]. \tag{21}$$

## G. Binomial parameter variance

The binomial variance can be computed exactly as

$$\begin{aligned}\mathrm{Var}_N\big[x_i(\lambda)\big] &= \frac{1}{N}\sum_i^N \left[\frac{p\lambda + x_i}{1+\lambda}\right]^2 - \left[\frac{p\lambda + \langle x_j \rangle}{1+\lambda}\right]^2 \\ &= \frac{1}{N}\sum_i^N \left[2\frac{x_i p\lambda + x_i^2}{(1+\lambda)^2} - 2\frac{\langle x_j \rangle p\lambda - \langle x_j \rangle^2}{(1+\lambda)^2}\right] \\ &= \frac{\langle x^2 \rangle - \langle x_j \rangle^2}{(1+\lambda)^2} = \frac{\sigma_x^2}{(1+\lambda)^2}.\end{aligned} \tag{22}$$

## H. Multinomial growth rate variance

The variance of a function, $\gamma(x)$, of a random variable, $x$, is given generally by the Taylor expansion of that function [62]. It is written as

$$\begin{aligned}\mathrm{Var}_N\big(\gamma[x_i(\lambda)]\big) &= \gamma'\big[\big\langle x_i(\lambda)\big\rangle\big]\mathrm{Var}_N\big[x_i(\lambda)\big] \\ &- \frac{\gamma''\big[\big\langle x_i(\lambda)\big\rangle\big]^2}{4}\mathrm{Var}_N^2\big[x_i(\lambda)\big] + \bar{T}^3,\end{aligned} \tag{23}$$

where primes denote differentiation with respect to $x$, and $\bar{T}^3$ are higher order terms that are only relevant at small times. The first and second-order derivatives of $\gamma$ are given by

$$\begin{aligned}\gamma'(x) &= \frac{p}{x} - \frac{1-p}{1-x} \\ \gamma''(x) &= -\left[\frac{p}{x^2} + \frac{1-p}{(1-x)^2}\right],\end{aligned} \tag{24}$$

and the variance term is given by

$$\mathrm{Var}_N\big[x_i(\lambda)\big] = \frac{\sigma_x^2}{(1+\lambda)^2}. \tag{25}$$

The growth rate variance after small times is given by

$$\begin{aligned}\mathrm{Var}_N\big(\gamma[x_i(\lambda)]\big) &= \left[\frac{p}{\bar{x}} - \frac{1-p}{1-\bar{x}}\right]\frac{\sigma_x^2}{(1+\lambda)^2} \\ &+ \left[\frac{p}{\bar{x}^2} + \frac{1-p}{(1-\bar{x})^2}\right]\left[\frac{\sigma_x^2}{(1+\lambda)^2}\right]^2,\end{aligned} \tag{26}$$

where for brevity, $\bar{x} \equiv \big\langle x_i(\lambda)\big\rangle$.
